# Supplementary material for: “Alone, there is nobody”: A qualitative study of the lived experience of loneliness in older men living with HIV
Source: PLoS One. 2023 Apr 14;18(4):e0277399. doi: 10.1371/journal.pone.0277399 (PMC10104292; doi:10.1371/journal.pone.0277399)
Supplement: Attachment — Submitted filename: Final_ResponseToReviewers_Sept2022.docx [file pone.0277399.s001.docx]

Response to Reviewers

Please find attached our revised manuscript entitled **“Alone, there is nobody”: A qualitative study of the lived experience of loneliness in older men living with HIV”**. We thank the reviewers for their helpful comments and appreciate their overall positive assessment of this manuscript. We have undertaken revisions to address their comments, which we detail point-by-point, below.

Reviewer #1

***Comment:*** The authors commence with a broad definition of loneliness as ‘as a negative feeling in response to a lower quantity or quality of social support than desired’, though there is little engagement with the broad sociology of loneliness. Instead, the authors rely on public health emphasises on health outcomes. Indeed, as the authors note at various points, an inductive thematic analysis proceeds from what the data says about loneliness, not how data fits into pre-existing frameworks. However, in order to make the claim that the psycho-social aspects of loneliness, especially in relation to stigma, the authors must engage with this significant body of sociological work. Else, more care needs to be taken to ensure that the definition employed is not assumed in participants’ descriptions. Otherwise, I question which definition of loneliness was employed by research participants and how was loneliness narratives identified by researchers.

**Response**: We did not want to make any a priori assumption about the nature of loneliness, so we have opted for the 2^nd^ option. In the introduction, we have included the definitions used in many of the quantitative studies reviewed; but have clarified that our focus was on the *experiences* of loneliness *in our population* from their first-person perspective. In addition, the comment prompted critical reflexivity on what language we might be imposing on the categories. We then reviewed the themes, being attentive to keep them consistent with the language and terms used by the participants in the quotes of those thematic sections. This led to changes, such as the elimination of the theme “survival mode” which had been under “living with” (i.e. survival is not really living at all), and integrating the content into the theme “multiple losses”. Another example, is the integration of “Seeking fun” into “Pursuing interests ‘worth living for’” which taken from the a citation of a participant as closer to the participants’ own meaning.

***Comment:*** Relatedly, given the outsized role of stigma, the authors need to engage more with the literature to avoid taking stigma as a priori. For example, I read both interpersonal and anticipated/internalised stigma in some of the participants’ accounts. In the limitations, the authors were at pains to point to correlation not causation, yet causation is implied throughout. A more careful engagement with the theory(ies) of stigma is needed to ensure that the layering of stigma over experiences and coping with loneliness to robust.

**Response**: We are a bit unclear about the meaning of ‘outsized’. Our focus was on understanding loneliness from the perspective of the participants. Engagement with the different theories of stigma could, indeed, be an interesting angle to take with the data in the future. However, at the time that the study was developed and conducted, our focus was on understanding the experiences of loneliness from participants’ first-person perspectives. We recognized that the use of the concept of stigma in the findings was not grounded within a theoretical framework of stigma and thus removed it from the findings. The findings are grounded in the language and experiences of the participants, and we discuss those findings relative to research on aging, HIV and stigma.

***Comment*:** I applaud the authors for their innovation in their approach to member checking (usually confined to sending participants copies of transcripts or emergent findings). However, the dyadic nature of the second phase needs more careful attention. Akin to focus groups, this phase needs attention to both the thematic AND interactionalist content of those sessions. These would have a synergistic element, and I wonder how this interaction led to the refinement of the first theme, but also the suggestions for future interventions. Also, it was unclear whether some/all the quotes were derived from the first or second phase (e.g., page 13 quote). This needs to be clear upfront. How was data presented in pages 2? Anonymous? No quotes?

**Response**: All of the quotes were derived from Stage 1 unless specified as being from Stage 2. We believe the Reviewer might mean, “How was the data presented in Stage 2?” (i.e., not page 2?). If this is the case, only the themes from Stage 1 were presented to the participants in Stage 2 and, thus, were anonymous with no quotes included. The methods have been elaborated to further describe Stage 2 and how the themes were refined.

***Comment:*** Associated with this, I had some questions around method. The authors mention what I relate as the coproduction of knowledge in the first phase, whereby narratives are presented and then subjectively interpreted by the interviewer. This is evident in the emphasis on significant, rather than cumulative, losses. How did the authorship team manage this, noting of course that this was listed as a limitation? More detail, then, is needed on an explanation of the benefits/challenges of narrative-phenomenology, as this is the first time I have heard of it together (conventionally, these methodologies are considered separate). What is this approach, other than a narrative-derived approach to analysing significant events? And if simply this, what makes this approach different to phenomenology?

**Response**: This study did not use a participatory approach, which might have emphasized a co-production of knowledge with participants as co-researchers. Instead, we used Charmaz’ more phenomenological approach to grounded theory, supplemented by a narrative-phenomenological conceptual framework to focus data collection and events on “significant experiences” as described by participants. Narrative phenomenology is a conceptual framework that emerged from longitudinal, multiple-perspective ethnographies of African American children with disabilities and their families. It is a distinct approach to phenomenology that is person-, event- and discursive centered as described in Cheryl Mattingly’s (2010) Paradox of Hope. It is a theoretical framework grounded in hermeneutic philosophy (e.g., Gadamer, Ricouer) and narrative theories (e.g., see Mattingly’s thorough discussion in Mattingly & Garro, 2000 and Mattingly, 2010). This attention to narrative as enacted by particular persons focuses attention on how, through actions—including verbal action—persons create significant experiences or “events” (see Mattingly, 1998). As *both* narrative *and* phenomenological, this theoretical or conceptual framework can guide both how one structures questions to help elicit stories (e.g., see for example, Mattingly & Lawlor 2000) as well as identify significant experiences during analysis to understand what might be most at stake. As a theoretical framework it focuses attention to a particular mode of experience rather than experience in general, while the hermeneutic resources focus the interpretive process on understanding the meaning of that experience from the first-person perspective of those who share those experiences. We have tried to clarify that this provides focused attention on significant *experiences—*that, in this research project was to understand those related to loneliness—the value of which can be either positive or negative.

A significant experience, by its nature, is singular rather than additive (e.g., see also Jackson’s 2010 definition of event in his existential anthropology). Certainly, hermeneutic approaches require reflexivity as Gadamer clearly describes in *Truth and Method,* since interpretive acts always involve the experiences of the interpreter. At the same time, reflexivity can also help support rigour that can help researchers untangle how their own experiences shape interpretation, while a focus on co-production may or may not lead to reflexivity on how the context of research, itself, or situatedness of the academic researcher relative to participant might contribute unequally whose voice has more weight in the co-production of knowledge. In addition, Phase 2, also served—as the reviewer noted—to help support (or not) the interpretation of the data.

***Comment:*** The relationship of this sub study to the larger +BHN needs further explanation. Why were only 6 lonely and 4 non-lonely selected? Given the distinction between loneliness or no-loneliness, which quotes are attributed to which self-reported loneliness scale? How did +BHN findings also influence this analysis? How many were in original sample? How were they recruited? If by direct invitation, how was perceived coercion managed? There also needs to be further explanation of the scales selected for participant inclusion (e.g., why work, age, living with another, why not volunteering experience, intimate relationships, etc. included?).

**Response:** All participants were drawn from the parent study, +BHN which has over 800 participants. The selection criteria were the response to a question in the parent study about loneliness, age over 50, self-reported male gender, self-reported white ethnicity and living in Montreal where the interviews took place. Information about specific age, work, living situation etc... was volunteered by the participants during the interview, but were not part of the selection process and was not queried directly. The participant demographics were included for interest but did not play a part in the selection of the participants or in data saturation. The sample size of 10 was determined by data saturation. While we selected participants who reported feeling lonely vs not at the time of the questionnaire, all had experienced loneliness at some point and as such contributed to the understanding of loneliness. Participants were invited by the research coordinator that they had already been in contact with for the parent study. The ethical aspects of recruitment for the parent study and the current sub-study were reviewed by the institutional research ethics board.

In terms of the data analysis, the quantitative study on loneliness in the +BHN cohort was being done on data that had previously been collected and the team that was doing that analysis was separate from the team doing this study. The two studies were meant to stand separately with the qualitative one having more details on the lived experience and how participants would want services to be targeted to help.

***Comment:*** I found the table presentation very difficult, a simple X denoting yes or no would have been easier to read.

**Response**: We have modified the tables as per the reviewer’s suggestion

***Comment:*** Stage 1 questions are very broad, but also an emphasis on ‘you’, rather than relational questions. I would be more comfortable with an explanation of how these questions were developed, and what role of the interviewer in prompts, follow-ups etc*.*

**Response**: We had cited two references that describe in detail the types of questions used to help elicit stories (Mattingly & Lawlor, 2000; Davidson, 2000). Both sources describe in-depth how a focus on eliciting others’ experiences from their own perspective is invaluable, particularly for persons whose experiences are often marginalized. The prompts, in general, are to elicit more details about those experiences. We are unclear what the concern is about this approach as it is also supported by more recent attention to what Miranda Fricker calls, testimonial epistemic justice (the importance of listening to the stories of those who are often silenced) and hermeneutic epistemic justice (the importance of the persons’ own hermeneutic or symbolic resources). We are also not clear what might be a “relational” question.

Reviewer #2

***Comment:*** The definition of loneliness is very brief and refers only to social support. The references cited to support this definition offer a much more comprehensive definition of loneliness related to the absence, or limitations, of social networks, the quality of social relationships, trust and solidarity. It would be good if some of these themes are brought into the definition here as it is possible for people to be lonely even if they don’t require emotional or practical support as strictly defined.

Also, in paragraph two, loneliness is described as a feeling. Without wanting to be too finicky, I wonder if that is accurate? While I appreciate that loneliness is a feeling, and that emotions reflect and reinforce social processes, the policy and programmatic response to loneliness referred to above (e.g., the UK government response) is really a response to loneliness as a social phenomenon. Again, a more detailed definition of loneliness might tease this out

**Response**: We have replaced the definition that was in the introduction by a broader statement, including how loneliness is defined within quantitative studies and noting their datedness. We have acknowledged that we allowed the definition of loneliness *in our population* to emerge from the narratives as is seen in the results section called “experiences of loneliness”

***Comment*:** In this same paragraph, it would be helpful to provide a brief outline of the reasons why loneliness is associated with stigma. Have the studies referred to provided insight into this?

**Response:** We did not bring in the concept of stigma as a priori because it was emphasized in the literature at the time that the study was developed and conducted, and it mainly appeared as one element in cross-sectional analyses. Our focus was on understanding loneliness from the perspective of the participants. To lessen this confusion, we also kept close to the language of the participants in the findings. We then discuss those findings with more recent research that directly addresses stigma. We appreciate both Reviewer 1 and 2s highlighting the need to separate out mention of stigma, until the discussion and also added over half a dozen new references to the discussion.

*Comment:* Methods. The section under the subheading ‘Qualitative approach’ is a little hard to follow. In general, it covers too much ground for a short paragraph and some concepts raised need more detail. For example, the statement ‘As specified by Charmaz (2006) in her approach to grounded theory, our findings are based on the experiences of our research participants as they are interpreted by the researcher’. More details on the subjective position of the researcher as this informed their approach to data analysis is needed to build from this statement. Also, more details on narrative-phenomenology and its person-centered, event-centered focus on experience is needed. Explain what this is and how it has been applied to this research.

Response:

More details were provided on narrative phenomenology across the methods section and a detailed explanation of this method is provided to reviewer 1, above. More details were also given about the positionality of the researchers : “The theoretical codes were then discussed by the authors who each drew from different epistemologies related to their different backgrounds in psychodynamic psychotherapy, anthropology, psychology and phenomenology to provide further rigour to the analysis.”

***Comment:*** Discussion. There are a few places where the meaning is not clear to the reader. For example, “The interviewer remembered being told about the multiple losses, but the stories of individual loss resonated with her more as they were closer to her own experience.” I think this means that it was difficult for the interviewer to understand the impact of multiple losses based on personal experience, but it reads as thought the researcher is reflecting on some specific elements of her life which would need explaining for the reader to understand how this shaped the analysis.

**Response:** The manuscript was read closely, to shift passive voice to active voice to add to the clarity of the findings. In addition, all findings were more fully and directly grounded in the actual language of the participants, with removal of the researcher experience (author 1) which is more indirect.

***Comment:*** The point that most people cannot fathom multiple losses and thus provide empathy needs some deeper consideration with respect to existing literature. I imagine this topic has been addressed in studies on loneliness and ageing given cumulative loss of friends and partners is very much an experience of ageing. I also know that there was work on grief and loss among gay men in relation to multiple losses from AIDS conducted in the 1990s that may be worth looking at here.

**Response:** We thank the reviewer for this comment as it helped us bring in the details to better describe how the “multiple losses” (described in findings) extend beyond the losses experienced during the HIV/AIDS epidemic. We then also discuss these losses in terms of their “accumulation” over time.

***Comment:*** The discussion could be a bit shorter and more focused on implications rather than describing each element of the findings. A general edit should do this, however.

**Response:** Thank you for your comment, we have integrated more recent references on intersectionality and stigma, activities and housing to bring out the implications of the findings.
